# Therapeutic properties of *Inonotus obliquus* (Chaga mushroom): A review

**DOI:** 10.1080/21501203.2023.2260408

**Published:** 2023-10-20

**Authors:** Phoebe Tee Yon Ern, Tang Yin Quan, Fung Shin Yee, Adeline Chia Yoke Yin

**Affiliations:** a School of Biosciences, Faculty of Health & Medical Sciences, Taylor’s University, Subang Jaya, Selangor, Malaysia; b Department of Molecular Medicine, Faculty of Medicine Building, University of Malaya, Kuala Lumpur, Malaysia

**Keywords:** Medicinal mushroom, *Inonotus obliquus*, therapeutic properties, anti-inflammatory, anticancer, anti-diabetic

## Abstract

*Inonotus obliquus*, also known as Chaga, is a medicinal mushroom that has been used for therapeutic purposes since the sixteenth century. Collections of folk medicine record the application of Chaga for the treatment of diseases such as gastrointestinal cancer, diabetes, bacterial infection, and liver diseases. Modern research provides scientific evidence of the therapeutic properties of *I. obliquus* extracts, including anti-inflammatory, antioxidant, anticancer, anti-diabetic, anti-obesity, hepatoprotective, renoprotective, anti-fatigue, antibacterial, and antiviral activities. Various bioactive compounds, including polysaccharides, triterpenoids, polyphenols, and lignin metabolites have been found to be responsible for the health-benefiting properties of *I. obliquus*. Furthermore, some studies have elucidated the underlying mechanisms of the mushroom’s medicinal effects, revealing the compounds’ interactions with enzymes or proteins of important pathways. Thus, this review aims to explore available information on the therapeutic potentials of *Inonotus obliquus* for the development of an effective naturally sourced treatment option.

## Graphical abstract

**Figure:**
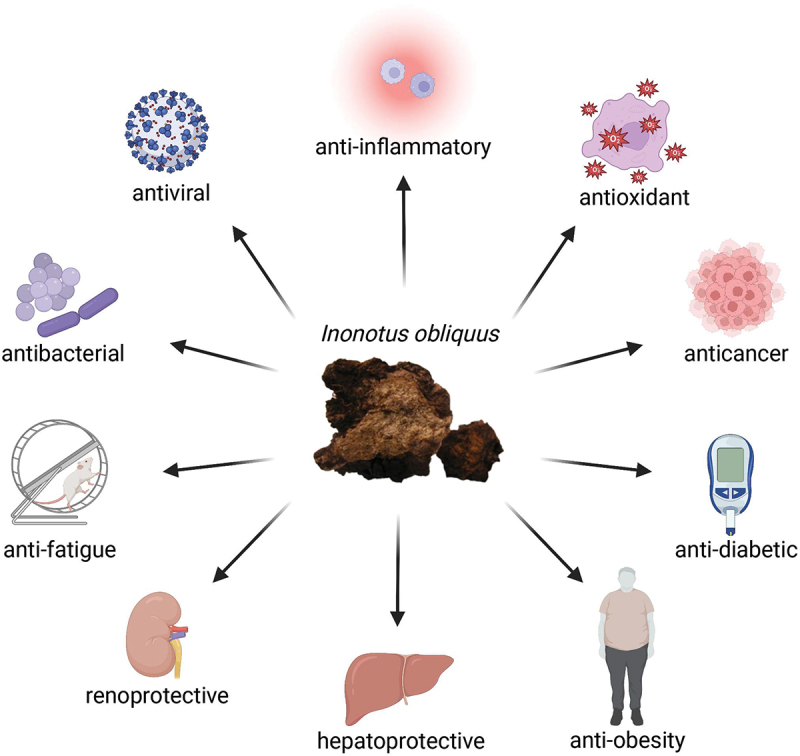

## 1. Introduction

**Figure 1.**
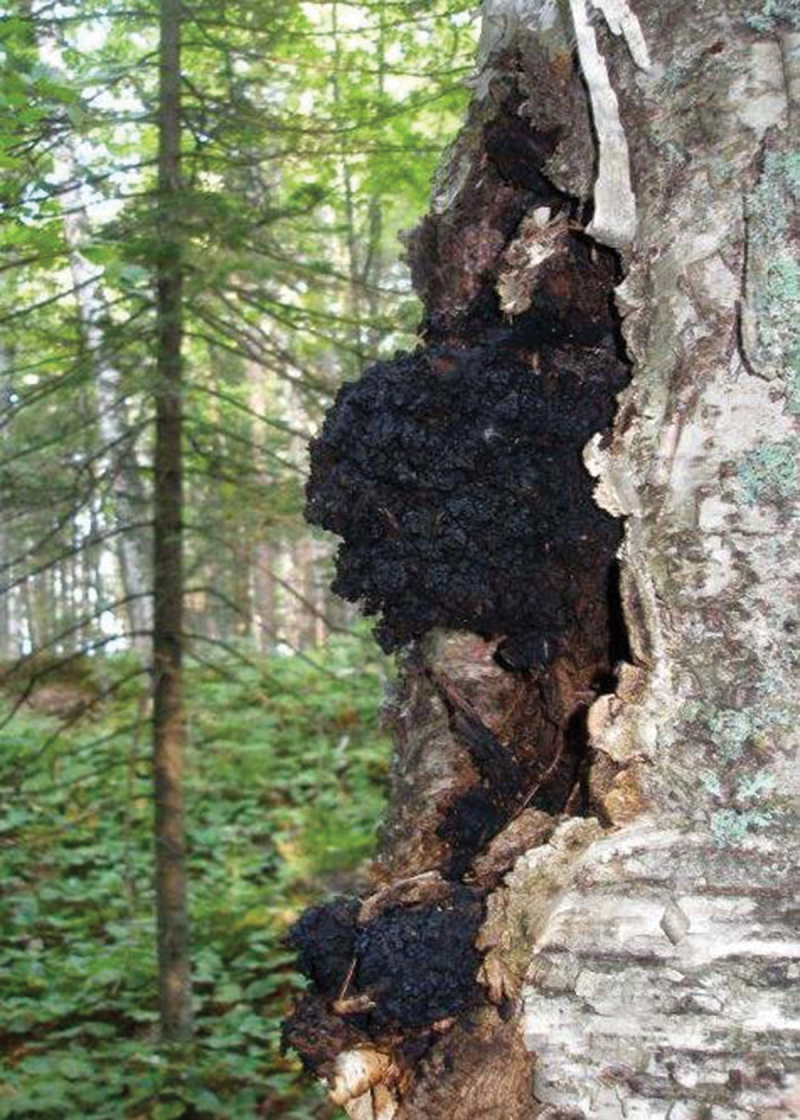
*Inonotus obliquus* sterile basidiome or conk naturally occurring on *Betula papyrifera* (Bal et al. 2019).

*Inonotus obliquus* (Chaga mushroom) is a black parasitic fungus that inhabits the trunk of birch trees (*Betula* spp.), as seen in Figure 1, in temperate and boreal regions of the northern hemispheres (Dai 2012). This tree disease fungus is commonly seen in Europe, Asia, and North America (Zheng et al. 2010). On infected stems of the trees, the fungus forms sterile conks with a charcoal-like appearance, with a dark brown pulp formed by interwoven mycelial mass (Lee et al. 2008). Growth of this fungi is mainly distributed in higher latitudes, where extremely low temperatures engender the fungi’s slow growth (Lu et al. 2021). Owing to this, infected trees can grow for 30–80 years without signs of decline, reaching sizes of more than 50 cm in diameter on old trees (Zheng et al. 2010).

Due to poverty and lack of access to scientific medicine, rational premises of folk medicine are based on natural substances for the alleviation of disease symptoms (Szychowski et al. 2021). Since the 16^th^ century, *I. obliquus* has been used as a folk medicine in Siberia, Russia, and other occidental countries. It has been used to treat diseases such as gastrointestinal cancer, cardiovascular diseases, and diabetes with minimal toxicity (Niu et al. 2016). People in Siberia used the fungus as a traditional medicine for the treatment of helminthic infections, tuberculosis, and liver diseases (Saar 1991). In North and Middle Russia, Chaga concentrated on tinctures used for the prophylaxis and treatment of gastric disorders and cancers (Shashkina et al. 2006). Chaga made into tea or concentrates is also widely consumed in Russia and Korea for its health-benefiting properties (Rhee et al. 2008).

**Figure 2.**
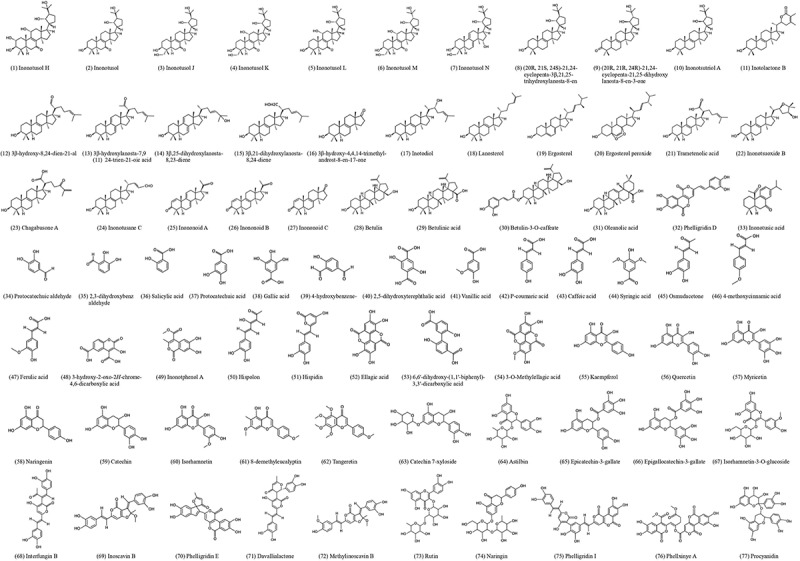
Structures of bioactive compounds isolated from *Inonotus obliquus.*

Upon chemical analysis of *Inonotus obliquus*, an extensive variety of bioactive substances have been found (Liu et al. 2014), where their structures can be found in Figure 2. Secondary metabolites that have been isolated from *I. obliquus* include polysaccharides, polyphenols, lanostane-type triterpenoids, and inotodiol (Duru et al. 2019). These compounds have been regarded as the active constituents that give rise to a myriad of health-promoting functions, indicating the high medicinal value of Chaga mushroom (Song et al. 2013). Furthermore, it is well tolerated by patients, non-toxic, and possesses virtually no contraindications for medicinal applications (Shashkina et al. 2006), further enhancing *I. obliquus*’ suitability for utilisation as a therapeutic agent.

Currently, most of the commercial Chaga products utilise wild-harvested *I. obliquus* sterile conks from birch trees, as wild sterile conks contain beneficial bioactive compounds mentioned previously, where some compounds, such as betulinic acid, originate from the *Betula* spp. host tree itself (Thomas et al. 2020). The rising popularity of Chaga products has resulted in increased commercial harvesting practices, raising concerns over the sustainability of Chaga harvesting. Although an assessment of the sustainability of commercial harvesting revealed the exceedingly abundant biological resources of *I. obliquus* with no risk of over-harvesting, this study is over a decade old (Pilz 2004) leaving us with the unknown impact of increased Chaga harvesting. However, artificial cultivation of *I. obliquus* on potato dextrose agar has been successful, where the content of bioactive compounds lanosterol, ergosterol, and inotodiol were observed to be similar to wild *I. obliquus* (Sun et al. 2011). Inoculations of birch trees have also been attempted and found to be successful, with harvestable conks forming as soon as three years post-inoculation (Silvan and Sarjala 2017; Miina et al. 2021).

This review aims to present relevant findings and explore the biological properties of *I. obliquus* mushroom and its potential therapeutic purposes.

## 2. Bioactive compounds and their therapeutic properties of *Inonotus obliquus*

### 2.1. Triterpenoids

Triterpenoids are a group of compounds found in natural products such as *I. obliquus*, synthesised from a common C30 precursor squalene (Mukherjee 2019). Variations in structural arrangements and ring closures have led to more than 4,000 identified cyclic triterpenoids, possessing a wide range of biological activities (Ghosh 2020), such as those listed in Table 1.

**Table 1.** Triterpenoids isolated from *Inonotus obliquus* and their biological activities.

Compound	Subject	Biological activity	Reference
Inonotusol H–N (**1–7**)	BV2 microglial cells	Inhibition of LPS-induced nitric oxide (NO) production	Kou et al. (2021)
(20R, 21S, 24S)-21,24-cyclopenta-3β,21,25-trihydroxylanosta-8-en (**8**) (20R, 21R, 24R)-21,24-cyclopenta-21,25-dihydroxylanosta-8-en-3-one (**9**) Inonotsutriol A (**10**) Inotolactone B (**11**)	Inhibition kinetics and molecular stimulations	Inhibitory activity against α-glucosidase	Chen et al. (2021)
3β-hydroxy-8,24-dien-21-al (**12**)	C57BL/6 primary macrophages	Reduction of LPS + IFNγ-induced NO production	Wold et al. (2020)
	RAW 264.7 macrophages	Inhibition of LPS-induced NO production and NF-κB luciferase activation	Ma et al. (2013)
	*In vitro* activity assay Kun Ming hyperuricemic mice	Inhibit xanthine oxidase activity	Luo et al. (2021)
	Prostatic carcinoma and breast carcinoma cells (PC3 and MDA-MB-231)	Cytotoxicity against cancer cells	Ma et al. (2013)
	Human fibrosarcoma cells (HT1080)	Inhibition of cancer cell invasion	Ryu et al. (2017)
	Human lung cancer cells (A549)	Cytotoxicity against cancer cells	Baek et al. (2018)
3β-hydroxylanosta-7,9(11),24-trien-21-oic acid (**13**)	Molecular docking	Inhibit xanthine oxidase	Yong et al. (2018)
	Inhibition kinetics and molecular stimulations	Inhibitory activity against α-glucosidase	Chen et al. (2021)
3β,25-dihydroxylanosta-8,23-diene (**14**)	Human fibrosarcoma cells (HT1080)	Inhibition of cancer cell invasion	Ryu et al. (2017)
3β,21-dihydroxylanosta-8,24-diene (**15**)	*In vitro* activity assay Kun Ming hyperuricemic mice	Inhibit xanthine oxidase activity	Luo et al. (2021)
	Human fibrosarcoma cells (HT1080)	Inhibition of cancer cell invasion	Ryu et al. (2017)
3β-hydroxy-4,4,14-trimethyl- androst-8-en-17-one (**16**)	Inhibition kinetics and molecular stimulations	Inhibitory activity against α-glucosidase	Chen et al. (2021)
Inotodiol (**17**)	C57BL/6 primary macrophages	Reduction of LPS + IFNγ-induced NO production	Wold et al. (2020)
	RAW 264.7 macrophages	Inhibition of LPS-induced NO production and NF-κB luciferase activation	Ma et al. (2013)
	*In vitro* activity assay Kun Ming hyperuricemic mice	Inhibit xanthine oxidase activity	Luo et al. (2021)
	Prostatic carcinoma and breast carcinoma cells (PC3 and MDA-MB-231)	Cytotoxicity against cancer cells	Ma et al. (2013)
	Prostatic carcinoma, gastric adenocarcinoma, and breast carcinoma cells (PC3, AGS, and MCF-7)	Inhibit cancer cell proliferation	Kim et al. (2020)
	Human fibrosarcoma cells (HT1080)	Inhibition of cancer cell invasion	Ryu et al. (2017)
	Cervical cancer cells (HeLa)	Induction of apoptosis	Zhang et al. (2019)
	Methionine-choline-deficient (MCD) diet-treated C57BL/6J mice OA-induced LO2 hepatocytes	Ameliorate lipid accumulation	Peng et al. (2022)
Lanosterol (**18**)	RAW 264.7 macrophages	Reduction of LPS + IFNγ-induced NO production	Ma et al. (2013)
	*In vitro* activity assay Kun Ming hyperuricemic mice	Inhibit xanthine oxidase activity	Luo et al. (2021)
	Human fibrosarcoma cells (HT1080)	Inhibition of cancer cell invasion	Ryu et al. (2017)
	Methionine-choline-deficient (MCD) diet-treated C57BL/6J mice OA-induced LO2 hepatocytes	Ameliorate lipid accumulation	Peng et al. (2022)
Ergosterol (**19**)	RAW 264.7 macrophages	Reduction of LPS + IFNγ-induced NO production	Ma et al. (2013)
	Prostatic carcinoma and breast carcinoma cells (PC3 and MDA-MB-231)	Cytotoxicity against cancer cells	Ma et al. (2013)
Ergosterol peroxide (**20**)	RAW 264.7 macrophages	Reduction of LPS + IFNγ-induced NO production	Ma et al. (2013)
	Prostatic carcinoma and breast carcinoma cells (PC3 and MDA-MB-231)	Cytotoxicity against cancer cells	Ma et al. (2013)
	Colorectal cancer cells (HCT116, HT-29, SW620, and DLD-1)	Inhibit cancer cell proliferation and clonogenic colony formation	Kang et al. (2015)
	AOM/DSS-treated C57BL/6 mice	Suppress colonic tumour growth	
	Microcystin-induced Balb/c mice	Hepatoprotective effect	Ishfaq et al. (2022)
Trametenolic acid (**21**)	C57BL/6 primary macrophages	Reduction of LPS + IFNγ-induced NO production	Wold et al. (2020)
	RAW 264.7 macrophages	Inhibition of LPS-induced NO production and NF-κB luciferase activation	Ma et al. (2013)
	Molecular docking	Inhibit xanthine oxidase	Yong et al. (2018)
	Prostatic carcinoma and breast carcinoma cells (PC3 and MDA-MB-231)	Cytotoxicity against cancer cells	Ma et al. (2013)
	Prostatic carcinoma, gastric adenocarcinoma, and breast carcinoma cells (PC3, AGS, and MCF-7)	Inhibit cancer cell proliferation	Kim et al. (2020)
	Human lung cancer cells (A549)	Cytotoxicity against cancer cells	Baek et al. (2018)
	Methionine–choline-deficient (MCD) diet-treated C57BL/6J mice OA-induced LO2 hepatocytes	Ameliorate lipid accumulation	Peng et al. (2022)
	C57BLKS/db mice with C57BL/6 mice as control	Ameliorate serum renal function indicator levels, reduce renal histological alterations, exert anti-fibrotic effect on kidneys	Duan et al. (2022)
Inonotsuoxide B (**22**)	Hepatic stellate cells (HSC-T6)	Anti-fibrotic activity by suppressing hepatic stellate cells	Jin et al. (2022)
Chagabusone A (**23**)	Human lung cancer cells (A549)	Cytotoxicity against cancer cells	Baek et al. (2018)
Inonotusane C (**24**)	Inhibition kinetics and molecular stimulations	Inhibitory activity against α-glucosidase	Chen et al. (2021)
	Molecular docking	Bind to SARS-CoV-2 spike protein receptor-binding domain	Basal et al. (2021)
Inononoid A–C (**25–27**)	Inhibition kinetics and molecular stimulations	Inhibitory activity against α-glucosidase	Chen et al. (2021)
Betulin (**28**)	C57BL/6 primary macrophages	Reduction of LPS + IFNγ-induced NO production	Wold et al. (2020)
	Molecular docking	Inhibit xanthine oxidase	Yong et al. (2018)
	*In vitro* activity assay Kun Ming hyperuricemic mice	Inhibit xanthine oxidase activity	Luo et al. (2021)
	Inhibition kinetics and molecular stimulations	Inhibitory activity against α-glucosidase	Chen et al. (2021)
Betulinic acid (**29**)	C57BL/6 primary macrophages	Reduction of LPS + IFNγ-induced NO production	Wold et al. (2020)
	Prostatic carcinoma, gastric adenocarcinoma, and breast carcinoma cells (PC3, AGS, and MCF-7)	Inhibit cancer cell proliferation	Kim et al. (2020)
	Inhibition kinetics and molecular stimulations	Inhibitory activity against α-glucosidase	Chen et al. (2021)
	3T3-L1 mouse adipocytes	Reduce lipid accumulation	Kim et al. (2019)
	Molecular docking	Bind to SARS-CoV-2 spike protein receptor-binding domain	Basal et al. (2021)
Betulin-3-O-caffeate (**30**)	C57BL/6 primary macrophages	Reduction of LPS + IFNγ-induced NO production	Wold et al. (2020)
Oleanolic acid (**31**)	*In vitro* activity assay Kun Ming hyperuricemic mice	Inhibit xanthine oxidase activity	Luo et al. (2021)
Phelligridin D (**32**)	*In vitro* assays	Attenuate reactive oxygen species and malondialdehyde levels, enhance superoxide dismutase and catalase activity	Li et al. (2021)
Inonotusic acid (**33**)	Molecular docking	Inhibit xanthine oxidase	Yong et al. (2018)

#### 2.1.1. Anti-inflammatory

Lanostane triterpenes (**1–7**) and triterpenoids (**12, 17, 21, 28, 29, 30**) extracted from *I. obliquus* inhibited nitric oxide (NO) production by lipopolysaccharide (LPS)-induced BV2 microglial cells and LPS + interferon gamma (IFNγ)-activated C57BL/6 primary macrophages, respectively (Wold et al. 2020; Kou et al. 2021). Further investigations using western blot and molecular docking analysis revealed that the reduction of NO generation could be attributed to the inhibitory activities of inonotusols I and L (**2, 5**) on LPS-induced inducible nitric oxide synthase (iNOS) expression and their strong interactions with iNOS protein (Kou et al. 2021). Ma et al. (2013) also reported significant inhibition of NO and NF-κB luciferase activation by triterpenoids (**12, 17–21**), thus giving rise to the observed anti-inflammatory activities of the isolated compounds.

#### 2.1.2. Antioxidant

Triterpenoids (**13, 21, 28, 33**) also displayed antioxidant effects through a strong inhibitory effect on liver xanthine oxidase activity, hence decreasing the generation of reactive oxygen species (ROS) (Yong et al. 2018). The triterpenoids (**12, 15, 17, 18, 28, 31**) could bind with the free enzyme more tightly than the enzyme-substrate complex, where this inhibitory interaction attenuates inflammation found in hyperuricemic mice (Luo et al. 2021). Phelligridin D (**32**) also contains antioxidant properties, capable of attenuating ROS and MDA, elevating SOD and CAT activity, and enhancing Nrf2 capacity for the promotion of ARE transcription (Li et al. 2021). Using siRNA interference, it was observed that phelligridin D was unable to reduce high ROS and MDA levels in cells transfected with Nrf2-siRNA, thus suggesting that phelligridin D-mediated protection against oxidative stress involves the activation of Nrf2.

#### 2.1.3. Anticancer

In a study assessing the anticancer abilities of five fractions (ethanol, petroleum ether, ethyl acetate, butanol, and water) extracted from *I. obliquus* on prostatic carcinoma cell line PC3 and breast carcinoma cell line MDA-MB-231, the petroleum ether extract was reported to have the highest cytotoxicity against PC3 and MDA-MB-231. This observation can be explained by the high triterpenoid content (**12, 17, 19–21**) of the petroleum ether fraction (Ma et al. 2013), as triterpenoids isolated by Kim et al. (2020) (**17, 21, 29**) also displayed anti-proliferative activity on PC3 and other cancer cell lines, AGS and MCF-7, in a dose-dependent pattern. Besides the petroleum ether extract, the ethyl acetate fraction also possessed relatively high inhibitory rates on the cell lines, where further analysis revealed that the main bioactive compounds of the two fractions were triterpenes ergosterol peroxide (**20**) and trametenolic acid (**21**) (Ma et al. 2013).

Apart from the abovementioned cancer types, ergosterol peroxide (**20**) also inhibited colorectal cancer cell proliferation in a concentration-dependent manner and substantially decreased both anchorage-dependent and anchorage-independent colony formation in HCT116, HT-29, SW620, and DLD-1 cells (Kang et al. 2015). Fluorescence activated cell sorting (FACS) analysis revealed that ergosterol peroxide stimulated apoptosis in HCT116 and HT-29 cells, represented by an increase in annexin V-positive or propidium iodide-positive cells. Furthermore, western blot analysis showed a decrease in uncleaved caspase-3 and an increase in cleaved poly (ADP-ribose) polymerase after ergosterol peroxide treatment, demonstrating the pro-apoptotic activities of ergosterol peroxide. Reduced nuclear levels of β-catenin protein were observed following treatment with ergosterol peroxide, which consequently led to suppression of c-Myc, cyclin D1, and cyclin-dependent protein kinase 8 (CDK-8) levels in the colorectal cell lines. Furthermore, microscopic observation of tissue sections showed suppression of colonic tumour development in AOM/DSS-treated mice after ergosterol peroxide treatment (Kang et al. 2015). The above observations suggest ergosterol peroxide isolated from *I. obliquus* as a potential chemotherapeutic agent.

Investigations on the invasion of human fibrosarcoma HT1080 cells through matrigel-coated filters demonstrated that triterpenes, namely 3β-hydroxylanosta-8,24-dien-21-al (**12**), inotodiol (**17**), and lanosterol (**18**), exert significant anti-invasive activities on the cancer cells (Ryu et al. 2017). Triterpenoids (**12**, **21**, **23**) isolated from *I. obliquus* fruiting bodies also exhibit cytotoxicity to the human lung cancer cell line A549 (Baek et al. 2018). Fascinatingly, the compounds were still capable of decreasing the cell viability of human adenocarcinoma cell lines with p53 mutations or null phenotype. This indicates that the cytotoxic activities of the extracts against human lung cancer cell lines were not attributable to p53-related pathways, but instead to the direct activation of caspase 3 (Baek et al. 2018). On the contrary, a lanostane-type triterpenoid named inotodiol (**17**) was demonstrated to exhibit antitumor activities by inducing apoptosis in HeLa cells through the p53-dependent pathway (Zhang et al. 2019).

#### 2.1.4. Anti-diabetic and anti-obesity

Enzymatic assay and inhibition kinetics analysis revealed that triterpenoids isolated from *I. obliquus* (**8–11, 13, 16, 24–29**) exert significant inhibitory activity against the α-glucosidase brush border enzyme, demonstrating their anti-diabetic properties (Chen et al. 2021). Furthermore, betulinic acid (BA) (**29**), a pentacyclic triterpenoid found in *I. obliquus*, was reported to possess anti-obesity activity in the HFD-induced obese mouse model (Kim et al. 2019). With no difference in caloric intake in the different groups of mice, the body weights of BA-treated mice were 10% lower than the untreated group, along with alleviation of obesity-associated dysregulation of serum lipid, insulin, and leptin. RT-PCR revealed enhanced mRNA expressions of genes regulating energy expenditure and decreased expressions of enzymes involved in triglyceride synthesis in BA-treated 3T3-L1 adipocytes, implying the effective protection of *I. obliquus* isolated betulinic acid against obesity.

#### 2.1.5. Hepatoprotective

Ishfaq et al. (2022) reported that the aqueous extract of *I. obliquus* (IOAE) is able to prevent microcystin-induced hepatic injury. They observed that IOAE restored levels of liver function indicators in MC-LR-treated mice, prevented MC-LR-induced oxidative stress by maintaining glutathione and catalase levels, and prevented histopathological damage of liver cells generated by microcystin. Molecular docking studies have revealed the possible molecular mechanism involved with the effects of IOAE, which is through the interaction of NF-κB-NIK with ergosterol peroxide (**20**) (Ishfaq et al. 2022).

Three *I. obliquus* constituents, including inotodiol (**17**), lanosterol (**18**), and trametenolic acid (**21**) also possess protective activity against non-alcoholic fatty liver disease (NAFLD) by exerting anti-lipid deposition effects, reversal of liver weight loss, reduction of liver triglyceride content, and restoration of dysregulated alanine transaminase (ALT) and aspartate aminotransferase (AST) levels (Peng et al. 2022). Upon further investigations, it was found that the protective effects of the extracts are via regulation of the FXR/SHP/SREBP-1c pathway (farnesoid X receptor/small heterodimer partner/sterol regulatory element-binding protein-1c), hence giving rise to their anti-NAFLD and hepatoprotective capabilities.

Another compound isolated from *I. obliquus*, inonotsuoxide B (**22**), was found to possess anti-fibrotic activity as they suppressed protein expression of α-smooth muscle actin (α-SMA) and type I collagen, reduced α-SMA mRNA expression induced by platelet-derived growth factor-BB (PDGF-BB), and activated the phosphatidylinositol 3-kinase/protein kinase B (PI3K/Akt) and extracellular signal-regulated kinase (ERK) signalling pathways, thus inhibiting the viability and activation of PDGF-BB-stimulated hepatic stellate cells (HSC-T6) to protect against hepatic fibrosis (Jin et al. 2022).

#### 2.1.6. Renoprotective

The renoprotective effects of trametenolic acid (TA) (**21**) extracted from *I. obliquus*, were observed in Duan et al. (2022) study using C57BL/6 mice and C57BLKS/db mice. TA was found to be capable of ameliorating serum blood urea nitrogen (BUN), creatinine, and urine albumin levels as well as reducing expansion of glomerular mesangial matrix and collagen deposition, hence indicating the restoration of renal function and alleviation of renal damage. Moreover, a significant boost in nephrin and podocin protein expression levels after treatment with TA, while immunohistochemical analysis detected a reduction in collagen III and fibronectin expression levels, suggesting the reversal of diabetic nephropathy-induced podocyte damage and fibrosis.

Besides TA, BA (**29**) also exhibited anti-fibrotic activity on adenine diet-induced chronic kidney disease (CKD) in rats (Sharma et al. 2017). Serum analysis revealed that BUN, creatinine, uric acid, serum cystatin C, and neutrophil gelatinase associated lipocalin (NGAL) levels were found to be lowered in BA-treated rats compared to control CKD groups. Furthermore, kidney histopathological changes, transforming growth factor beta (TGF-β), connective tissue growth factor (CTGF), fibronectin, collagen type I, and hydroxyproline levels were also significantly attenuated after BA treatment, hence indicating BA’s nephroprotective and anti-fibrotic effects.

#### 2.1.7. Antiviral

Apart from the previously mentioned bioactivities of triterpenoids, *in silico* studies revealed promising binding affinities of terpenoid compounds isolated from *I. obliquus* to the SARS-CoV-2 spike protein receptor-binding domain, where the best-scoring terpenoid is inonotusane C (**24**) at −7.8 kcal/mol (Basal et al. 2021). Furthermore, it was found that inonotusane C (**24**) and BA (**29**) bound to a location that is in close proximity to the ACE2 binding pocket on the SARS-CoV-2 spike protein, thus potentially affecting the viral recognition and invasion of the host cell.

### 2.2. Polysaccharides

#### 2.2.1. Anti-inflammatory

Another important extract is *I. obliquus* polysaccharide (IOP), which was observed to greatly reduce the mRNA expression of interleukin (IL)-17 and IFN-γ while exerting an upregulating effect on IL-4 and IL-10 expression (Chen et al. 2019). A reduction in tumour necrosis factor-α (TNF-α), IFN-γ, IL-1β, IL-4, and IL-6 expression in *Toxoplasma gondii*-infected macrophages by IOP was also demonstrated through ELISA and RT-PCR (Yan et al. 2021). Further immunocytochemistry analysis revealed that the inhibition of the inflammatory response is mediated by the prevention of NF-κB p65 translocation from the cytoplasmic space into the nucleus (Yan et al. 2021). This was similar to the results of Sang et al. (2022), where it was found that IOP reduces overexpression of inflammatory mediators through inhibition of the over-phosphorylation of proinflammatory transcription factor NF-κB p65 and inhibitor IκBα in *Toxoplasma gondii* infected cells. These findings suggest that polysaccharide extracted from *I. obliquus* is able to downregulate inflammatory mediators production and promote the generation of anti-inflammatory processes to ameliorate inflammation-related diseases such as colitis (Mishra et al. 2012).

#### 2.2.2. Antioxidant

Polysaccharides extracted from *I. obliquus* displayed scavenging activities for DPPH-radicals, hydroxyl radicals (Du et al. 2013; Hu et al. 2016), superoxide anion (Huang et al. 2012), and H_2_O_2_ (Du et al. 2013) in a concentration-dependent manner. In addition, using the ferric-reducing power assay, it was demonstrated that the IOP fractions possessed reducing powers in a dose-dependent pattern (Du et al. 2013; Wang et al. 2018). IOP was also reported to decrease Keap1 levels and increase Nrf2 levels (Han et al. 2019), hence allowing the promotion of an antioxidant transcription program (Baird and Yamamoto 2020).

The antioxidative effects of subcritical water extracted-IOP (SWE-IOP) and hot water extracted-IOP were compared based on their SOD-like activities, DPPH scavenging activities, and hydroxyl radical scavenging activities (Yuan et al. 2017). The results showed that SWE-IOP exhibited stronger antioxidative effects than hot water extracted-IOP, suggesting that subcritical water extraction could be a more advantageous method for extracting polysaccharides from *I. obliquus*.

It is noteworthy that the antioxidant properties of the IOP can be affected by both physical and chemical modifications. In particular, acid or alkali hydrolysis, thermal treatment, and ultrasonic treatment are some of the methods that can be used to modify the physicochemical properties of IOP and enhance its antioxidant abilities. Acetylated IOP (Ac-IOP) displayed the highest antioxidant capabilities in both assays for ferric-reducing power and liver lipid peroxidation inhibition, compared to sulphated-IOP and carboxymethylated-IOP (Ma et al. 2012). In addition, Zhang et al. (2013) reported that thermal treated polysaccharides (Th-IOP) and ultrasonic treated polysaccharides (Ul-IOP) exerted the strongest antioxidant activities on ferric-reducing power and liver lipid peroxidation inhibition assays. Thus, these studies suggest the potential therapeutic utilisation of Ac-IOP, Th-IOP, and Ul-IOP as potent antioxidants.

#### 2.2.3. Anticancer

Besides the anti-inflammatory and antioxidant properties, IOP has also been found to induce apoptotic cell death in treated human lung cancer cells (LLC1 and A549 cell lines) through liver kinase B1 (LKB1) activation of adenosine monophosphate-activated protein kinase (AMPK) and reduction of mitochondrial membrane potential (MMP) (Jiang et al. 2020).

The findings of Lee et al. (2017) differ from those of the earlier study, as they did not observe significant apoptotic activity of the tested IOP concentration range (0 to 100 μg/mL) on A549 cells. Nevertheless, in a chemotactic directional migration assay, IOP at 100 μg/mL was shown to inhibit A549 cell invasion by suppressing the levels of p-JNK (c-Jun N-terminal kinase) and p-AKT (protein kinase B) levels, as well as reducing the expression levels of matrix metalloproteinases (Lee et al. 2017). Furthermore, CCK-8 assay, cell scratch assay, transwell assay, and flow cytometry analysis revealed that IOP substantially reduced proliferation, migration, and invasion, and increased apoptosis, respectively, of MG-63 and U2OS osteosarcoma cells (Su et al. 2020). Western blot analysis showed inhibitory activity on the expression of related proteins in the protein kinase B/mammalian target of rapamycin (Akt/mTOR) signalling pathway by IOP, indicating that the antitumor effect of IOP against osteosarcoma is through hindering Akt/mTOR activation.

#### 2.2.4. Anti-diabetic

Various studies have found polysaccharides to be the main bioactive component of *I. obliquus* responsible for its anti-diabetic activities. Studies using the type 2 diabetes mellitus (T2DM) mice model have demonstrated that IOP improved insulin resistance, restored hepatic glycogen levels, ameliorated impaired glucose tolerance, and exerted antihyperglycemic effects (Wang et al. 2017; Chen et al. 2022). These observations are believed to be due to the upregulated levels of glucose transporter protein type-4 (GLUT4) expressions in adipose tissues and activation of PI3K/Akt signalling pathway (Wang et al. 2017).

IOP was also reported to be capable of significantly increasing glucose consumption of both wild-type HepG2 cells and insulin-resistant HepG2 cells (Xue et al. 2018; Liu et al. 2018). The promotive effect on glucose consumption by one of the polysaccharides (IOEP2) at concentrations of 40 μg/mL and 80 μg/mL was observed to be even higher than the commonly used hypoglycaemic drug metformin (Xue et al. 2018). Furthermore, enzymatic assay and inhibition kinetics analysis revealed that polysaccharides isolated from *I. obliquus* exert significant inhibitory activity against the α-glucosidase brush border enzyme (Dai et al. 2022), stronger than that of the oral hypoglycaemic agent acarbose (Liu et al. 2018), thus reducing postprandial hyperglycaemia.

Besides hyperglycaemia, another notable risk factor for the progression of diabetes is dyslipidaemia. Studies observed an exceptional relief of dysregulated lipid profiles in diabetic mice models after IOP treatment (Xu et al. 2021), demonstrating the potential of IOP as a promising source of treatment for diabetes and its complications.

#### 2.2.5. Anti-obesity

IOP has been reported to ameliorate obesity in a high-fat diet (HFD) mouse model through the upregulation of 19 miRNA involved in glucose metabolism and triglyceride metabolism (Table 2) (Yu et al. 2020).

**Table 2.** Phenols and flavonoids isolated from *Inonotus obliquus* and their biological activities/findings.

Compound	Molecular formula	Biological activity/findings	Reference
Protocatechuic aldehyde (**34**)	C_7_H_6_O_3_	Radical-scavenging activity and binding affinity to superoxide dismutase 1 enzyme	Hao et al. (2023)
		Radical-scavenging activity and protection against DNA damage	Hwang et al. (2016)
2,3-dihydroxybenzaldehyde (**35**)	C_7_H_6_O_3_	Major contributor of total antioxidant activity	Abu-Reidah et al. (2021)
Salicylic acid (**36**)	C_7_H_6_O_3_	Major contributor of total antioxidant activity	Abu-Reidah et al. (2021)
Protocatechuic acid (**37**)	C_7_H_6_O_4_	Major contributor of total antioxidant activity	Abu-Reidah et al. (2021)
		Radical-scavenging activity and protection against DNA damage	Hwang et al. (2016)
Gallic acid (**38**)	C_7_H_6_O_5_	Radical-scavenging and ferric-reducing activity	Wang et al. (2021)
4-hydroxybenzene-1,3-dioic acid (**39**)	C_8_H_6_O_5_	Radical-scavenging activity and protection against DNA damage	Hwang et al. (2016)
2,5-dihydroxyterephthalic acid (**40)**	C_8_H_6_O_6_	Major contributor of total antioxidant activity	Abu-Reidah et al. (2021)
		Radical-scavenging activity and binding affinity to superoxide dismutase 1 enzyme	Hao et al. (2023)
Vanillic acid (**41**)	C_8_H_8_O_4_	Major contributor of total antioxidant activity	Abu-Reidah et al. (2021)
P-coumaric acid (**42**)	C_9_H_8_O_3_	Radical-scavenging and ferric-reducing activity	Wang et al. (2021)
		Major contributor of total antioxidant activity	Abu-Reidah et al. (2021)
		Anticancer activity	Wang et al. (2015)
Caffeic acid (**43**)	C_9_H_8_O_4_	Radical-scavenging and ferric-reducing activity	Wang et al. (2021)
		Major contributor of total antioxidant activity	Abu-Reidah et al. (2021)
Syringic acid (**44**)	C_9_H_10_O_5_	Radical-scavenging activity and protection against DNA damage	Hwang et al. (2016)
		Major contributor of total antioxidant activity	Abu-Reidah et al. (2021)
		Radical-scavenging activity and binding affinity to superoxide dismutase 1 enzyme	Hao et al. (2023)
Osmundacetone (**45**)	C_10_H_10_O_3_	Major contributor of total antioxidant activity	Abu-Reidah et al. (2021)
		Radical-scavenging activity and binding affinity to superoxide dismutase 1 enzyme	Hao et al. (2023)
		Radical-scavenging activity and protection against DNA damage	Hwang et al. (2016)
4-methoxycinnamic acid (**46**)	C_10_H_10_O_3_	Major contributor of total antioxidant activity	Abu-Reidah et al. (2021)
Ferulic acid (**47**)	C_10_H_10_O_4_	Radical-scavenging activity	Xu et al. (2016)
		Major contributor of total antioxidant activity	Abu-Reidah et al. (2021)
		Anticancer activity	Wang et al. (2015)
3-hydroxy-2-oxo-2H-chrome-4,6-dicarboxylic acid (**48**)	C_11_H_6_O_7_	Radical-scavenging activity and protection against DNA damage	Hwang et al. (2016)
		Radical-scavenging activity and binding affinity to superoxide dismutase 1 enzyme	Hao et al. (2023)
Inonotphenol A (**49**)	C_12_H_10_O_6_	Radical-scavenging activity	Chang et al. (2022)
Hispolon (**50**)	C_12_H_12_O_4_	Major contributor of total antioxidant activity	Abu-Reidah et al. (2021)
Hispidin (**51**)	C_13_H_10_O_5_	Major contributor of total antioxidant activity	Abu-Reidah et al. (2021)
Ellagic acid (**52**)	C_14_H_6_O_8_	Major contributor of total antioxidant activity	Abu-Reidah et al. (2021)
6,6’-dihydroxy-(1,1′-biphenyl)-3,3′-dicarboxylic acid (**53**)	C_14_H_10_O_6_	Radical-scavenging activity and protection against DNA damage	Hwang et al. (2016)
3-O-Methylellagic acid (**54**)	C_15_H_8_O_8_	Major contributor of total antioxidant activity	Abu-Reidah et al. (2021)
Kaempferol (**55**)	C_15_H_10_O_6_	Radical-scavenging and ferric-reducing activity	Wang et al. (2021)
Quercetin (**56**)	C_15_H_10_O_7_	Radical-scavenging and ferric-reducing activity	Wang et al. (2021)
		Major contributor of total antioxidant activity	Abu-Reidah et al. (2021)
Myricetin (**57**)	C_15_H_10_O_8_	Major contributor of total antioxidant activity	Abu-Reidah et al. (2021)
Naringenin (**58**)	C_15_H_12_O_5_	Major contributor of total antioxidant activity	Abu-Reidah et al. (2021)
Catechin (**59**)	C_15_H_14_O_6_	Radical-scavenging activity and binding affinity to superoxide dismutase 1 enzyme	Hao et al. (2023)
Isorhamnetin (**60**)	C_16_H_12_O_7_	Major contributor of total antioxidant activity	Abu-Reidah et al. (2021)
8-demethyleucalyptin (**61**)	C_18_H_16_O_5_	Radical-scavenging activity and binding affinity to superoxide dismutase 1 enzyme	Hao et al. (2023)
Tangeretin (**62**)	C_20_H_20_O_7_	Radical-scavenging and ferric-reducing activity	Wang et al. (2021)
Catechin 7-xyloside (**63**)	C_20_H_22_O_10_	Radical-scavenging and ferric-reducing activity	Wang et al. (2021)
Astilbin (**64**)	C_21_H_22_O_11_	Radical-scavenging and ferric-reducing activity	Wang et al. (2021)
Epicatechin-3-gallate (**65**)	C_22_H_18_O_10_	Radical-scavenging activity	Xu et al. (2016)
		Antioxidant; increased accumulation through lignocellulose degradation	Zhao et al. (2021)
Epigallocatechin-3-gallate (**66**)	C_22_H_18_O_11_	Radical-scavenging activity	Xu et al. (2016)
		Antioxidant; increased accumulation through lignocellulose degradation	Zhao et al. (2021)
Isorhamnetin-3-O-glucoside (**67**)	C_22_H_22_O_12_	Radical-scavenging and ferric-reducing activity	Wang et al. (2021)
Interfungin B (**68**)	C_23_H_18_O_8_	Radical-scavenging activity and binding affinity to superoxide dismutase 1 enzyme	Hao et al. (2023)
Inoscavin B (**69**)	C_24_H_20_O_8_	Antioxidant; increased accumulation through wheat-straw culture	Zhao et al. (2021)
Phelligridin E (**70**)	C_25_H_14_O_10_	Radical-scavenging activity and binding affinity to superoxide dismutase 1 enzyme	Hao et al. (2023)
Davallialactone (**71**)	C_25_H_20_O_9_	Antioxidant; increased accumulation through wheat-straw culture	Zhao et al. (2021)
Methylinoscavin B (**72**)	C_25_H_22_O_8_	Radical-scavenging activity and binding affinity to superoxide dismutase 1 enzyme	Hao et al. (2023)
Rutin (**73**)	C_27_H_30_O_16_	Antioxidant; increased accumulation through lignocellulose degradation	Zhao et al. (2021)
Naringin (**74**)	C_27_H_32_O_14_	Radical-scavenging activity	Xu et al. (2016)
		Antioxidant; increased accumulation through lignocellulose degradation	Zhao et al. (2021)
Phelligridin I (**75**)	C_33_H_20_O_13_	Radical-scavenging activity and binding affinity to superoxide dismutase 1 enzyme	Hao et al. (2023)
Phellxinye A (**76**)	C_30_H_20_O_16_	Radical-scavenging activity	Chang et al. (2022)
Procyanidin (**77**)	C_30_H_26_O_13_	Radical-scavenging and ferric-reducing activity	Wang et al. (2021)

DNA sequencing of caecal microbiota revealed that the ameliorative effects of IOP on obesity are through modification of the microbiota metabolism, particularly an increase in butyrate-production-associated bacteria *Lactobacillus* and *Bacteroidales* S24-7, as well as short-chain fatty acid-producing bacteria *Holdemanella* and *Ruminococcaceae_UCG-014* (Yu et al. 2020). Furthermore, IOP can significantly reduce serum total cholesterol (TC), triglycerides (TG), and low-density lipoprotein-cholesterol (LDL-C) contents, while increasing high-density lipoprotein-cholesterol (HDL-C) content both *in vitro* in oleic acid-induced HepG2 cells and *in vivo* in mice fed with a high-fat diet (Yang et al. 2021; Lin et al. 2023). A successful decrease in the weight gain of mice induced by a high-fat diet was also observed after 10 weeks of IOP treatment (Yang et al. 2021).

In addition, lipid synthesis-related genes, sterol regulatory element-binding protein-1C (SREBP-1C), acetyl-coenzyme A carboxylase (ACC), and fatty acid synthase (FAS), were found to be lower in HFD-treated-C57BL/6J mice that received IOP intervention as compared to untreated groups (Yang et al. 2021). Instead, IOP treatment was observed to reverse the downregulated expressions of adenosine monophosphate-activated protein kinase (AMPK) in obese mice (Yang et al. 2021) and increased cholesterol 7 alpha-hydroxylase (CYP7A1) expressions (Lin et al. 2023), which is an enzyme involved in the main pathway for cholesterol removal from the body (Wang et al. 2018).

Oligosaccharides isolated from *I. obliquus* also provided protection against hyperlipidaemia. Kunming mice were fed a high-fat diet (HFD) for 1 week, then allowed to eat and drink freely with *I. obliquus* oligosaccharide (IOP-2A) administered by gavage in designated groups (Wu et al. 2021). Biochemical analysis of mice serum showed that IOP-2A is able to ameliorate dyslipidaemia by decreasing TC, TG, and LDL-C levels while increasing HDL-C concentrations. Moreover, the variation trend of mice body weight over the course of 8 weeks indicated that IOP-2A is able to reduce the extent of HFD induced-weight gain, hence suggesting IOP-2A as a promising source of anti-obesity treatment.

#### 2.2.6. Renoprotective

A study conducted on streptozotocin (STZ) + advanced glycation end product (AGE)-treated renal tubular LLC-PK1 cells revealed that IOP treatment can prevent STZ+AGEs-induced renal cell glucotoxicity and exert anti-fibrotic activity (Chou et al. 2016). In addition, analysis of differentially treated STZ-injected C57BL/6 mice found that IOP is able to attenuate and restore histopathological changes in the renal cortex, including the integrity of glomerular capsules and a number of glomerular mesangial cells. NF-κB and TGF-β expressions were significantly reduced in a concentration-dependent manner, thus suggesting that the observed renoprotective activities are partly attributed to the inhibition of the NF-κB/TGF-β pathway.

#### 2.2.7. Anti-fatigue

Studies have found that the Chaga mushroom is able to exert anti-fatigue effects in several studies using animal models. Xiuhong et al. (2015) employed a swimming-to-exhaustion experimental model to evaluate the anti-fatigue activities of *Inonotus obliquus* polysaccharides on Kunming mice, where results indicated that the swimming time to exhaustion in IOP-treated groups was significantly longer than in untreated mice. Blood sample analysis of mice showed that blood lactate and blood urea nitrogen were substantially reduced in IOP-treated mice as compared to the untreated control group, suggesting that IOP postpones the rise in blood lactic acid levels and postpones the onset of physical fatigue symptoms. Moreover, observation of liver and muscle tissues through light microscopy demonstrated a significantly higher glycogen content in IOP-treated groups than in the control group in a dose-dependent manner.

Another study reported other possible mechanisms of IOP that contribute to the improvement of fatigue in mice models (Zhang et al. 2020). RT-PCR analysis detected higher GRAF1 (guanosine triphosphatase regulator associated with focal adhesion kinase-1) mRNA expression in the gastrocnemius muscles of IOP-treated mice. Furthermore, the determination of total integrated optical density revealed a decrease in 5-hydroxytryptamine (5-HT) expression in the brains of mice administered with IOP. Thus, IOP is not only able to delay the onset of physical fatigue but also possesses promising abilities to alleviate mental fatigue.

### 2.3. Phenols and flavonoids

#### 2.3.1. Antioxidant

Phenols and flavonoids are highly responsible for the antioxidant activity of *I. obliquus* extracts, where hydrophilic phenolics (**35–37, 40–47, 50–52, 54, 56–57, 60**) were found to be major contributors to the antioxidant activity of the extracts (Abu-Reidah et al. 2021). Phenolic compounds (**34, 40, 44–45, 48, 58–59, 61, 68, 70, 72, 75**) were also reported to possess robust radical-scavenging activities and binding affinities to the superoxide dismutase 1 (SOD1) enzyme, especially styrylpyranones polyphenols such as Phelligridin E (**70**), suggesting their role in enhancing antioxidant enzyme activities (Hao et al. 2023).

Through a comprehensive analysis of constituents separated from *I. obliquus* using DPPH, ABTS, and FRAP analysis, it was found that two compounds, phellxinye A (**76**) and inonotphenol A (**49**), have the strongest antioxidant activities (Chang et al. 2022). Further electronic analysis of phellxinye A using the highest occupied molecular orbital (HOMO) and lowest unoccupied molecular orbital (LUMO) revealed potential active sites vital for the antioxidant properties of phellxinye A, including the isocoumarin fragment and *cis* double-bond present in the compound’s structure.

Polyphenols isolated from *I. obliquus* (**38, 42–43, 55–56, 62–64, 67, 77**) through orthogonal experimentally optimised aqueous ethanol extraction and purified by macroporous resin purification showed strong DPPH radicals and hydroxyl radicals scavenging activity, along with ferric-reducing activity, thus making them a good source of natural antioxidants (Wang et al. 2021). The dose-dependent antioxidant properties of phenolic compounds were also demonstrated by Hwang et al. (2016), where all phenolics isolated (**34, 37, 39, 44–45, 48, 53**) exhibited radical scavenging activities and protection against DNA damage. DPPH radical-scavenging activities of tween-20 induced *I. obliquus* polyphenols were observed to increase with the rising content of flavonoids ferulic acid (**47**), epicatechin-3-gallate (**65**), epigallocatechin-3-gallate (**66**), and naringin (**74**), thus indicating a strong correlation between fermentation growth-associated generation of flavonoids and the antioxidative activities of *I. obliquus* extracts (Xu et al. 2016). Therefore, attempts have been made to increase the production of phenolics and flavonoids by *I. obliquus* through different treatment processes to enhance its antioxidant activities. For example, Zhao et al. (2021) reported that wheat straw culture increased the accumulation of inoscavin B (**69**) and davallialactone (**71**), while lignocellulose degradation enhanced the generation of flavonoids ECG (**65**), EGCG (**66**), rutin (**73**), and naringin (**74**), thereby augmenting *I. obliquus’* antioxidative properties.

#### 2.3.2. Anticancer

Lignin metabolites labelled as IOW-S-1 and IOW-S-2 [containing P-coumaric acid (**42**) and ferulic acid (**47**)], were isolated from *I. obliquus* through hot-water extraction followed by ethanol precipitation (Wang et al. 2015). Studies have proven that these IOW-S-1 and IOW-S-2 were able to noticeably decrease the cell viability of A549, Bel-7402, and LO2 cell lines in a concentration-dependent pattern, where anti-proliferation rates reached 75% at maximum concentrations. This observation is believed to be due to their inhibitory activity on NF-κB p65 translocation in LPS treated cells (Wang et al. 2015), which is an important signalling pathway involved in the pathogenesis of cancer (Xia et al. 2018), thus explaining their anti-proliferative effect.

#### 2.3.3. Hepatoprotective

Melanins from aqueous extract of *I. obliquus* also demonstrated hepatoprotective effects when administered into Sprague Dawley rats with carbon tetrachloride-induced liver damage (Parfenov et al. 2019). Melanin was able to minimise signs of liver tissue damage such as necrosis, fat accumulation, and steatosis, and alter the total protein, serum cholinesterase, gamma-glutamyl transpeptidase, total bilirubin, and unconjugated bilirubin to more normal levels. Although biochemical parameters, including alkaline phosphatase, did not return to completely normal levels seen in healthy animals, the biochemical values were more normalised as compared to Carsil, indicating the advantage of melanin over the common hepatoprotective drug.

### 2.4. Extracts

#### 2.4.1. Anti-inflammatory

Extracts isolated from *I. obliquus*, using methanol as an extraction solvent, were reported to decrease histamine-induced TNF-α in RAW 264.7 macrophages by more than 90% (Javed et al. 2019). Further evaluation of the methanolic extract in a mouse microcirculation model revealed its ability to reverse the reduced conducted vasodilation response commonly experienced during inflammation. Similarly, Alhallaf and Perkins (2022) also found that *I. obliquus* extracts, especially those obtained by the accelerated ethanol/water extraction method and hot water steeping of powdered Chaga, are capable of exerting significant suppression of NO, TNF-α, IL-6, and IL-1β generation in RAW 264.7 macrophages, thus highlighting the potential of *I. obliquus* extracts as anti-inflammatory agents.

#### 2.4.2. Anticancer

Extracts of *I. obliquus* obtained from liquid-state fermentation have also been found to possess anticancer properties by exerting anti-proliferative effect on the HCT-116 cell line through the activation of mitochondrial apoptotic pathway by upregulating mRNA expressions of pro-apoptotic genes (Bax, bad, and caspase-3) and increasing Bax/bcl-2 ratio (Tsai et al. 2017). In the same study, *I. obliquus* extracts were also observed to influence the cell cycle, enhancing expressions of proapoptotic genes (p53, p21WAF1/CIP1) and downregulating anti-apoptotic genes (CyclinD1). In addition, 14 days of orally administered methanolic extracts inhibited lung tumour formation and metastasis in C57BL/6 mice injected with B16F10 melanoma cells (Ryu et al. 2017). *I. obliquus* extracts given as Chaga infusions to mice models of Lewis lung carcinoma (LLC) growth led to a reduction of CD31-positive vascular endothelial cells, indicating that the extracts exhibit significant retardation of tumour development by decreasing tumour vascularisation (Arata et al. 2016). Continuous measurement of body temperature in the mice showed that the intake of *I. obliquus* extract prevents the drop in body temperature in LLC-implanted mice, thus suggesting the potential role of body temperature maintenance in tumour suppression.

#### 2.4.3. Anti-diabetic

*I*. *obliquus* extracts obtained by high-pressure water extraction method were orally administered to type 2 diabetic C57BL/6 mice at 250 mg/kg and 500 mg/kg to evaluate their anti-diabetic capabilities (Zhang et al. 2021). Administration of extracts at both concentrations led to amelioration of blood glucose and insulin resistance, enhanced liver glycogen and HDL-C, and reduced total cholesterol, triglyceride, and LDL-C, where the effects 500 mg/kg were reported to be comparable to that of metformin. The properties of the *I. obliquus* extracts were found to be via the regulation of PI3K/Akt and AMPK/ACC signalling pathways, giving rise to the observed hypoglycaemic and hypolipidemic effects.

Furthermore, *I. obliquus* treatment of HFD+STZ-induced diabetic mice at 150 mg/kg greatly ameliorated the pathological state of fatty liver and fatty degeneration of liver cells, decreasing lipid droplet accumulation in the liver (Chen et al. 2022). The pathological states of other affected organs in diabetic mice were also found to be improved by *I. obliquus* treatment, including the kidneys, pancreas, and colon (Chen et al. 2022; Ye et al. 2022). Other symptoms of diabetes mellitus, such as intestinal barrier dysfunction, can also be alleviated by *I. obliquus* treatment through upregulation of Ki-67, zonula occludens-1 (ZO-1), and mucin-2 (MUC2) expression (Su et al. 2022), showing the promising role of *I. obliquus* for the relief of diabetes and its consequent complications.

#### 2.4.4. Hepatoprotective

At low concentrations of 10 µg/mL, hot water extract from *I. obliquus* (IO-W) was reported to protect cultured hepatocytes from cytotoxic injury induced by tert-butyl hydroperoxide (t-BHP) by quenching free radicals (Hong et al. 2015). The hepatoprotective activity of IO-W was also observed through its suppression of t-BHP-induced cellular leakage of ALT, AST, and lactate dehydrogenase (LDH), as well as malondialdehyde (MDA) (Hong et al. 2015).

#### 2.4.5. Renoprotective

Chiang et al. (2023) reported renoprotective activities of ethanol-ethyl acetate extracts of *I. obliquus* on nephropathic mice, where the extracts were able to effectively reduce creatinine and blood urea nitrogen levels, improve glomerular atrophy and interstitial accumulation, and reduce TGF-β and α-SMA expression. Besides the restored serum parameters and histological changes, a study conducted on STZ-induced Sprague Dawley rats observed a significant decrease in urinary protein excretion in *I. obliquus*-treated rats as compared to the control groups, after 8 weeks of treatment (Zhang et al. 2022). Colour Doppler ultrasonography examinations of the left renal interlobar artery in the rat models revealed amelioration of deteriorated blood flow parameters after Chaga treatment (50 mg/kg and 100 mg/kg), along with a restoration of peak systolic velocity (PSV), mean velocity (MV), and end-diastolic velocity (EDV) levels. The above results thus suggest the robust protective and ameliorative effects of *I. obliquus* on kidney histological architecture and function.

#### 2.4.6. Anti-bacterial

Both inhibitory and bactericidal activities of ethanolic and aqueous extracts of *I. obliquus* against all tested bacterial strains were observed, including both Gram-positive and Gram-negative bacteria, where *Staphylococcus aureus* and *Bacillus cereus* were the most sensitive to the extracts (Glamočlija et al. 2015). In addition, Milyuhina et al. (2021) found that although aqueous extracts of *I. obliquus* displayed antibacterial activity, the activity of the extracts was enhanced upon microwave radiation treatment. This observation can be attributed to the enhanced phenolic content of the extracts upon exposure to ultrahigh frequency radiation (Papoutsis et al. 2016), thus increasing the effects against microflora.

#### 2.4.7. Antiviral

Evaluation of the antiviral activities of four *I. obliquus* water extract fractions on hepatitis C virus (HCV)-infected porcine embryo kidney cell (SPEV) culture demonstrated that the extracts possess virucidal activity towards HCV, protective effects of SPEV cells from the HCV-induced pathogenic effects, and the ability to lead to reduction or complete absence of infective viruses in culture medium specimens 48 h after infection (Shibnev et al. 2011).

Extracts of *I. obliquus* also exhibit anti-herpetic activity on herpes simplex virus (HSV)-infected Vero cells (Polkovnikova et al. 2014; Nosik et al. 2020), which was found to be mediated by the prevention of HSV entry via action on viral glycoproteins, thus leading to prevention of viral-induced membrane fusion (Pan et al. 2013). In addition, *I. obliquus* extracts have antiviral effects on human immunodeficiency virus type 1 (HIV-1) in the lymphoblastoid cell culture MT-4 at concentrations of just 5.0 μg/mL, thus demonstrating its potent antiviral properties (Shibnev et al. 2015; Nosik et al. 2020).

Teplyakova et al. (2022) reported that aqueous extracts of *I. obliquus* also possess antiviral activities against SARS-CoV-2 *in vitro* on Vero E6 and Vero cell cultures, with an IC50 of 0.75 μg/mL for SARS-CoV-2 replication if prepared under optimal conditions. Hence, *I. obliquus* can be considered for its potential use as an effective treatment for COVID-19 virus infection.

## 3. Conclusions and future perspectives

The comprehensive information included in this review shows the extensive medicinal and therapeutic properties of Chaga mushroom, *I. obliquus*. Various extracts and compounds isolated from *I. obliquus* possess promising anti-inflammatory, antioxidant, antibacterial, and antiviral properties. In addition, *I. obliquus* has been found beneficial for the amelioration of various human ailments such as cancer, diabetes, obesity, hepatic disorders, renal diseases, and fatigue, thus making it an attractive natural alternative to pharmacological interventions.

Although the majority of the studies were able to reveal the pathways involved in the properties of *I. obliquus*, the exact underlying mechanisms for most of the mushroom’s health-benefiting effects are still not well understood. Furthermore, there is a need to ascertain if bioactive compounds of *I. obliquus* exert their therapeutic properties with the same efficacy when ingested in their free state and as part of food. It is also important to determine any potential antagonistic or synergistic interactions between *I. obliquus* and other compounds or drugs when taken together. Therefore, utilisation of the latest technologies and experimental procedures for the extensive studies of *I. obliquus* as a therapeutic agent should be conducted.
